# Semantic role labeling for protein transport predicates

**DOI:** 10.1186/1471-2105-9-277

**Published:** 2008-06-11

**Authors:** Steven Bethard, Zhiyong Lu, James H Martin, Lawrence Hunter

**Affiliations:** 1 Computer Science Department, University of Colorado at Boulder, Boulder, CO, USA; 2 National Center for Biotechnology Information, National Library of Medicine, Bethesda, MD, USA; 3 Center for Computational Pharmacology, University of Colorado School of Medicine, Aurora, CO, USA

## Abstract

**Background:**

Automatic semantic role labeling (SRL) is a natural language processing (NLP) technique that maps sentences to semantic representations. This technique has been widely studied in the recent years, but mostly with data in newswire domains. Here, we report on a SRL model for identifying the semantic roles of biomedical predicates describing protein transport in GeneRIFs – manually curated sentences focusing on gene functions. To avoid the computational cost of syntactic parsing, and because the boundaries of our protein transport roles often did not match up with syntactic phrase boundaries, we approached this problem with a word-chunking paradigm and trained support vector machine classifiers to classify words as being at the beginning, inside or outside of a protein transport role.

**Results:**

We collected a set of 837 GeneRIFs describing movements of proteins between cellular components, whose predicates were annotated for the semantic roles AGENT, PATIENT, ORIGIN and DESTINATION. We trained these models with the features of previous word-chunking models, features adapted from phrase-chunking models, and features derived from an analysis of our data. Our models were able to label protein transport semantic roles with 87.6% precision and 79.0% recall when using manually annotated protein boundaries, and 87.0% precision and 74.5% recall when using automatically identified ones.

**Conclusion:**

We successfully adapted the word-chunking classification paradigm to semantic role labeling, applying it to a new domain with predicates completely absent from any previous studies. By combining the traditional word and phrasal role labeling features with biomedical features like protein boundaries and MEDPOST part of speech tags, we were able to address the challenges posed by the new domain data and subsequently build robust models that achieved F-measures as high as 83.1. This system for extracting protein transport information from GeneRIFs performs well even with proteins identified automatically, and is therefore more robust than the rule-based methods previously used to extract protein transport roles.

## Background

Automatic semantic role labeling (SRL) is a natural language processing (NLP) technique that maps sentences to semantic representations, which can be useful for many NLP tasks (e.g. information extraction). With the advent of resources like FrameNet [1] and PropBank [2,3], this technique has had a flurry of activity in recent years. Much of this work has focused on the arguments of verbs, and because PropBank uses Wall Street Journal for its source documents, much of the work has been trained and evaluated on newswire text [4-9].

As a variety of research groups have reported success on these corpora, recent work has turned to transferring these results to different kinds of predicates and different genres of text. In this article, we show that automatic semantic role labeling can be transferred to the biomedical domain. Our goal is to accept as input sentences describing biological processes and infer structures like the following:

(1) [_PATIENT_ Bax] [_PREDICATE_ translocation] from the [_ORIGIN_ cytosol] to [_DESTINATION_ mitochondria] leads to the subsequent formation....

This predicate argument structure indicates, for example, that *Bax *has the PATIENT role in the *translocation *event, that is, *Bax *is the protein undergoing movement. In general, predicate argument structures characterize how different proteins and cellular components participate in biological events, and thus form the basis for understanding the text.

Semantic role labeling systems from newswire domains need some adjustments to perform well on biomedical text. Biomedical text differs widely from the newswire text commonly used to train SRL systems, both in the style of the written text and the predicates involved. Predicates in newswire text are typically verbs, for example:

(2) Four of the five [_PREDICATE(VERB)_ surviving] workers [_PREDICATE(VERB)_ have] asbestos-related diseases, [_PREDICATE(VERB)_ including] three with recently [_PREDICATE(VERB)_ diagnosed] cancer.

Biomedical text often prefers nominal predicates, and light verbs like *take *or *leave *[10], which typically have little semantic content of their own. Example 3 shows a typical sentence, where there are only two verbs, *left *and *abolished*, and the former serves only as a light verb supporting the predicate *unimpaired*.

(3) [_PREDICATE(NOUN)_ Truncation] of up to 44 C-terminal amino acids from the putatively cytoplasmic C-terminal hydrophilic domain left transport function [_PREDICATE(ADJ)_ unimpaired], but [_PREDICATE(NOUN)_ deletion] of the adjacent STAS (sulfate transporter anti-sigma factor antagonist) domain [_PREDICATE(VERB)_ abolished] function.

The predicates used in biomedical text are also quite unlike those of other corpora. Predicates like *endocytosis*, *exocytosis*, *internalize*, *traffic *and *translocate*, though common in texts describing protein transport, are completely absent from both the FrameNet and PropBank data.

Other researchers have explored the difficulties of adapting semantic role labeling technologies to new domains and have encountered the same two basic problems: differences in text style and differences in predicates. The CoNLL 2005 shared task [11] investigated semantic role labeling systems that were trained on the Wall Street Journal and tested on the Brown corpus. They found that "all systems experienced a severe drop in performance (about 10 F1 points)" when compared to their results on Wall Street Journal data, and attributed this drop to the poorer performance of sub-components like part-of-speech taggers and syntactic parsers. A similar performance drop was observed when semantic role labeling models were trained on nominal predicates. Pradhan et. al. [12] achieved an F-measure of only 63.9 when evaluating their models on nominal predicates from FrameNet and some manually annotated nominalizations from the Penn Chinese TreeBank. Jiang and Ng [13] achieved better results on the NomBank [14] corpus, but their F-measure was still only 72.7, more than 10 points below state of the art performance for verbs. Thus, these research efforts suggest that adapting semantic role labeling to biomedical text will offer some interesting challenges.

BIOSMILE [15] is the only SRL system of which we aware that targeted the biomedical domain. Our work significantly differs from BIOSMILE in both the data and algorithm that were used. The BIOSMILE system was trained on BioProp [16], a biomedical proposition bank semi-automatically annotated in the style of PropBank. BioProp, like other biomedical corpora of predicate argument structures, e.g. that of Kogan and colleagues [17], considered only verbs, annotating 30 biomedical verbs in 500 abstracts. In contrast, the corpus used in this work includes both verbal and nominal forms (e.g. both *translocates *and *translocation*) for a total of 86 unique predicates (34 unique lemmas) describing protein transport events. Because BioProp followed PropBank style, their semantic roles were only allowed to match full syntactic units, while our data includes multi-word roles that are smaller than a traditional TreeBank style syntactic unit, a necessity for handling nominal predicates. Because of these many differences in data, and to evaluate methods that did not rely on computationally expensive syntactic parses, we explored an alternative to the syntactic constituent approach used by BIOSMILE, and trained a word-chunking model on our corpus.

Our corpus consists of gene data from the National Library of Medicine (NLM). NLM began a Gene Indexing initiative on April 1, 2002, the goal of which was to link any article about the basic biology of a gene or protein to the corresponding entry in Entrez Gene [18], the National Center for Biotechnology Information's gene database. The result was an entry within Entrez Gene called a Gene Reference Into Function (GeneRIF) [19], which acts as an important textual source of the functional annotation of genes [20,21]. Our predicates and roles have been annotated over a subset of these GeneRIFs, for example:

(4) IRS-3 expression blocked glucose/IGF-1 induced [_PATIENT_ IRS-2] [_PREDICATE_ translocation] from the [_ORIGIN_ cytosol] to the [_DESTINATION_ plasma membrane].

GeneRIFs have been used in a variety of natural language processing projects on biomedical text, including projects to automate alerts for new findings [22] and to extract summaries of PubMed/MEDLINE records [23-26]. Most relevant to the research at hand is [27], which describes an information extraction system called OpenDMAP that combines pattern matching with domain specific ontologies to build applications capturing biomedical knowledge (e.g. protein transport).

We used data similar to that of [27], and focused in particular on predicates that describe protein transport. Protein transport is the biological process of moving proteins from one cellular component to another by various sorting mechanisms. For example, for extracellular signals to be transduced to the nucleus to activate specific genes, an essential step is translocating transcription factors into the nucleus. Understanding the mechanisms of protein transport has been a central theme in cell biology and has been studied for decades [28]. However, while natural language processing technologies have generally shown success in facilitating biomedical research [29-32], there is currently very little work that has focused on applying NLP techniques to the protein transport domain.

The GeneRIF data in our corpus were taken directly from the data used in developing and evaluating OpenDMAP (Open-source Direct Memory Access Parser) [27], an open-source, ontology-driven concept analysis engine. Like OpenDMAP, our system can be applied to automatically extract protein transport information from texts. The main difference is that OpenDMAP used knowledge either directly found in its ontology or indirectly obtained by external programs. Thus, its overall performance depended on the output accuracy of those third-party applications. As reported in [27], "A significant cause of errors in the OpenDMAP system as evaluated is incorrect identification of gene and protein names." For instance, a decrease of over 20% in recall of protein transport roles was reported in [33] when OpenDMAP was given automatically identified protein boundaries instead of human annotated boundaries. In contrast, only a slight (5%) decrease was shown in similar experiments presented in this work. We attribute the difference (5% vs. 20%) to the machine-learning models we employed, which were capable of recognizing proteins not found in the training data.

In addition to the ontology-driven approach of OpenDMAP, there are other fundamentally different IE approaches that are currently used extensively in the biomedical domain, including co-occurrence approaches, heuristic and rule-based approaches, and syntactic analysis and machine-learning approaches [34,35]. The semantic role labeling approach differs from these by focusing on the linguistically motivated semantic links between entities in a sentence. Such relations are common in most text, and have been shown to improve IE results dramatically [36], and so we chose to explore these methods here.

## Results

### Protein transport data analysis

We constructed a corpus of 837 GeneRIFs annotated with protein transport predicates and their AGENT, PATIENT, ORIGIN and DESTINATION roles. (See Methods section for details.) There were some interesting differences between this protein transport data and the more traditional semantic role data of resources like FrameNet and PropBank. Of course, as discussed above, there were a variety of predicates in the protein transport data which never occurred in the kind of newswire text that is common in FrameNet and PropBank. But in addition to these basic differences in predicate inventories, there were some structural differences in the data. About 85% of predicates in the protein transport data were nouns, with only 15% verbs. For comparison, the test data for FrameNet semantic role labeling in SensEval-3 [37] was about 40% nouns, 40% verbs and 20% adjectives. So protein transport predicates have a much greater bias toward nominal forms.

These nominal predicates introduce some additional challenges to semantic role labeling systems. First, many semantic relations are expressed by noun compounding, where many of the syntactic cues that were useful for verbs are unavailable. For example, there is often no subject/object distinction for nouns, so that a two-noun compound can be formed just as easily using the verbal equivalent's subject, object or prepositional object. So for example, given the phrase *The transporter translocates GLUT-4 to the nucleus*, paraphrases using the nominalization *translocation *could look like Example 5, Example 6 or Example 7.

(5) [_AGENT_ transporter] translocation

(6) [_PATIENT_ GLUT-4] translocation

(7) [_DESTINATION_ nuclear] translocation

Nominal predicates are also more difficult due to their mismatch with commonly available syntactic tree structures. The Penn TreeBank [38] gives a very flat structure to noun phrases. For example, the phrase *fatty acid transport protein translocation *would appear as a single NP with no internal structure, even though it contains an embedded nominal role, *fatty acid transport protein*:

(8) [NP [_PATIENT_ fatty acid transport protein] [_PREDICATE_ translocation]]

In fact, in our transport predicate data, about 20% of roles look like this – there is no single syntactic constituent that matches their boundaries. For comparison, in the Propbank data, only 2% of roles did not correspond to a single constituent, in the NomBank data 5% did not match, and in the FrameNet data 15% did not match. Note that this is likely a major difference between our corpus and the verb-oriented BIOSMILE corpus. Thus, the protein transport data imposes a number of challenges on semantic role labeling: a much greater bias towards nominal predicates, fewer syntactic cues to help identify the roles, and a large mismatch between the role boundaries and the syntactic constituent boundaries. These difficulties need to be addressed when designing a semantic role labeling method for protein transport data.

### Experimental results

For the purposes of our machine learning experiments, 200 GeneRIFs were selected at random from our protein transport corpus and reserved as the test set, to be used only for the final evaluation. The remaining 637 GeneRIFs were used to train machine learning models based on the word-chunking machine learning approach discussed in the Methods section. YamCha [39], our SVM-based machine learning algorithm, requires a number of different parameters to be specified: the cost of misclassification, the degree of the polynomial and the width of the feature window. To determine the best set of these parameters, we first ran a number of cross-validations on the training set, varying each parameter over a number of possible values, and checking the cross-validation performance. For the Word-Chunking and Protein-Transport models, the best cost was 10.0, the best polynomial degree was 2, and the best window size was 2 words before and after. For the Phrase-Chunking model, the best cost was 0.1, the best polynomial degree was 1, and the best window size was 2 words before and after.

These parameters were then used to train the models on the full 637 GeneRIFs in the training data. We trained models on the following feature sets:

#### Word-Chunking

The basic Word-Chunking Features of [40]. (See the Methods section for details.)

#### Phrase-Chunking

The Word-Chunking features plus the Phrase-Chunking Features derived from the phrase-chunking model of [40]. (See the Methods section for details.)

#### Protein-Transport

The Phrase-Chunking features plus the features inspired by the analysis of the protein transport data: the orthographic features, the MedPost part of speech tags, the protein BIO chunk labels, and the conjunction and coreference features. (See the Methods section for details.)

We evaluate these models in terms of precision, recall and F-measure:

$$\begin{array}{c} P=\frac{N_{correct}}{N_{predicted}}\\ R=\frac{N_{correct}}{N_{reference}}\\ F_{1}=\frac{2*P*R}{P+R} \end{array}$$

Precision is the number of roles the system identified correctly divided by the number of roles the system predicted. Recall is the number of roles the system identified correctly divided by the number of roles that were present in manually annotated data. *F*_1_-measure (often abbreviated simply as F-measure) is defined as the harmonic mean of precision and recall.

The task can also be viewed as a two step process in which boundaries are first identified and then role classes are labeled. Under this view, the models are evaluated in terms of unlabeled precision, unlabeled recall and labeled accuracy. Unlabeled precision and recall are just like precision and recall but ignore the label type (e.g. AGENT or PATIENT) and only check that the boundaries of the roles are correct. Labeled accuracy reports, for the roles whose boundaries were correctly identified, the percent that were assigned the proper label type.

Table 1 gives precision, recall, F-measure and labeled accuracy values for our models when evaluated on the 200 GeneRIFs reserved as our test set. The model trained using only the simple word-chunking features was able to achieve 79.7% precision and 64.4% recall, reasonably good results given that this model relied on only 5 simple features. Adding in all the features derived from the phrase-chunking model raised model performance up to 81.0% precision and 71.9% recall, indicating that our translation of the Hacioglu [40] phrase chunking features to word chunking features was effective. The model that incorporated all the protein transport features in addition to the word and phrase features achieved 87.6% precision and 79.0% recall, a more than 6% gain in both precision and recall over the best baseline model. Since some of the protein transport features were derived from manually annotated protein annotations, it was also useful to examine how using automatic protein annotations affected performance. The last row of Table 1 shows these numbers. Using the automatic protein annotations from ABNER resulted in less than a 1% drop in precision, but in almost a 5% drop in recall. These results were still higher than the baseline word-chunking and phrase-chunking models, but it was clear that the protein-based features were playing a strong role in the model, and having lower quality protein annotations resulted in lower quality semantic roles.

**Table 1:** Model performance by feature set.

	Labeled			Unlabeled		Labeled
			
	Precision	Recall	F-measure	Precision	Recall	Accuracy
Word-Chunking	79.7	64.4	71.3	80.1	64.7	99.6
Phrase-Chunking	81.0	71.9	76.2	81.9	72.7	98.9
Protein-Transport	87.6	79.0	83.1	87.9	79.2	99.7
Protein-Transport (ABNER)	87.0	74.5	80.3	87.3	74.8	99.7

This table shows precision, recall, F-measure and labeled accuracy statistics for semantic role labeling models trained on various feature sets. "ABNER" indicates the results when using the ABNER-identified proteins instead of the manually annotated ones.

## Discussion

The high performance of our final model indicated that existing semantic role labeling techniques can be adapted to domains such as protein transport by adding a few carefully chosen domain-relevant features. Performance dropped slightly when using automatically identified protein boundaries, but as automatic protein identification systems like ABNER improve, we should see similar improvement in the performance of our role labeler.

### Analysis by role type

One of the interesting characteristics of all our models was that when our models were able to find a role, they typically had little trouble identifying the type of that role – labeled accuracy was 98% and higher for all models. This high classification accuracy can probably be attributed to two factors. First, the annotation style dictated that the AGENT and PATIENT roles were always proteins, while ORIGIN and DESTINATION roles were always non-proteins. Thus while occasional confusions between, say, ORIGIN and DESTINATION might have been possible, confusions between, say, AGENT and DESTINATION should have been extremely unlikely. Second, these four roles, particularly in the protein transport domain, appear in a somewhat limited number of forms. For example, work on a related corpus suggested that only five OpenDMAP-style patterns were required for good performance [27]. As can be seen from our evaluation results, our machine learning model performed like a set of high precision patterns would have – with very few confusions, and with precision substantially higher than recall.

Thus, the main issue for our models was not in distinguishing one role from another, but in finding the roles in the first place. To get an idea of how difficult the different types of roles were to identify, we calculated precision, recall and F-measure on each role type for our best model, the model using the Protein-Transport feature set with manually annotated protein boundaries. The results are shown in Table 2. Our models found only one of the three AGENT examples in our testing data due to data sparsity issues – AGENT roles made up less than 1% of the roles in our protein transport predicates. AGENT roles were also harder because they tended to be further from the predicate. On the average, less than 50% of AGENT roles were within three words of the predicate, while more than 75% of PATIENT, ORIGIN and DESTINATION roles were within this window. Roles that were closer to the predicate were easier for our system to identify because they appeared within the word window our models considered during classification.

**Table 2:** Model performance by role type.

	Precision	Recall	F-measure	% of Roles
AGENT	100.0	33.3	50.0	0.8
PATIENT	86.5	74.4	80.0	51.7
ORIGIN	82.9	75.6	79.1	11.7
DESTINATION	90.3	87.7	89.0	35.8

This table shows precision, recall, F-measure split up by the type of role being identified. The final column indicates the percent of the total roles each role type accounted for. Performance numbers were calculated from the output of the Protein-Transport model when using manually annotated protein boundaries.

Our models performed best on DESTINATION roles, probably because DESTINATION roles appeared in fewer different forms. For example, the pattern *nuclear *<predicate> accounted for about 30% of all DESTINATION roles. To further elaborate on this kind of analysis, we calculated seen/unseen statistics for each role phrase. That is, we calculated separate precision, recall and F-measure values for the role phrases that appeared in both the training data and the test data, and for the role phrases that appeared only in the test data. Table 3 shows these statistics. Though all roles see some drop in performance from seen roles to unseen roles, the most dramatic drops are for AGENT roles, where unseen AGENT roles are *never *identified, and ORIGIN roles, where there is a 75 point drop in F-measure (from 90.4 to 15.4). As discussed above, the difficulties with AGENT roles are almost certainly due to their sparsity, but the difficulties with ORIGIN roles suggest this class of roles is intrinsically more difficult. These results indicate that some additional feature engineering may be required to better characterize ORIGIN roles. Fortunately, most ORIGIN roles were seen in the training data, and only 13.3% of ORIGIN roles are of this more difficult type.

**Table 3:** Model performance by seen vs. unseen.

	Precision	Recall	F-measure	%
Seen roles	97.7	88.9	93.1	60.1
Unseen roles	71.6	63.6	67.4	39.2

Seen AGENT roles	100.0	100.0	100.0	33.3
Unseen AGENT roles	100.0	0.0	0.0	66.7

Seen PATIENT roles	100.0	86.7	92.9	37.7
Unseen PATIENT roles	78.3	66.9	72.2	62.3

Seen ORIGIN roles	97.1	84.6	90.4	86.7
Unseen ORIGIN roles	14.3	16.7	15.4	13.3

Seen DESTINATION roles	96.5	91.6	94.0	86.2
Unseen DESTINATION roles	57.1	63.2	60.0	13.8

This table shows precision, recall, F-measure comparing role phrases which appeared in both the training and testing data ("seen" roles) to role phrases which appeared only in the testing data ("unseen" roles). Both overall results and results by role type are shown. The final column indicates the percent of the particular category of roles that were seen (or unseen). Performance numbers were calculated from the output of the Protein-Transport model when using manually annotated protein boundaries.

### Analysis of genre issues

To determine how well our additional features addressed the issues particular to the the biomedical genre, we checked model performance for some different predicate types. In particular, we examined how model performance varied with the part of speech of the predicate (nominal predicates are much more common in our data), and with the domain from which the predicate was drawn. Table 4 shows the results of this analysis. With just the basic Phrase-Chunking feature set, F-measure for verbal predicates, which make up only 20.8% of our data, is dramatically lower than for nominal predicates: 58.1 for verbs compared with 70.7 for nouns. Adding in the Protein-Transport features both increases overall performance and substantially reduces this disparity – the model achieves an F-measure of 80.6 for nominal predicates and an F-measure of 76.7 for verbal predicates. Thus, the Protein-Transport features help to address the difficulties of the increased number of nominal predicates.

**Table 4:** Model performance by predicate type.

	Feature Set	Precision	Recall	F-measure	% of Roles
Nominal predicates	Phrase-Chunking	78.8	63.0	70.7	79.2
Verbal predicates	Phrase-Chunking	64.3	52.9	58.1	20.8
Nominal predicates	Protein-Transport	86.2	75.8	80.6	79.2
Verbal predicates	Protein-Transport	88.5	67.6	76.7	20.8

Wall Street Journal predicates	Phrase-Chunking	74.0	62.7	67.9	34.3
GeneRIF-only predicates	Phrase-Chunking	77.3	60.7	68.0	65.7
Wall Street Journal predicates	Protein-Transport	87.0	79.7	83.2	34.3
GeneRIF-only predicates	Protein-Transport	86.3	72.1	78.6	65.7

This table shows precision, recall, F-measure split up by the predicate part of speech and domain. A predicate was considered to be in the Wall Street Journal domain if the word appeared anywhere in the Wall Street Journal section of the Penn TreeBank, and was considered a GeneRIF-only predicate otherwise. The second column indicates which model (which feature set) the performance numbers are for. The final column indicates the percent of roles accounted for by each part of speech and each predicate domain.

Analyzing general differences in text style from one domain to another is more difficult, but to approximate it, we considered two classes of predicates: predicates that occurred somewhere in the Wall Street Journal section of the Penn TreeBank (e.g. *delivery*, *move *and *released*), and predicates which were only observed in our GeneRIF data (e.g. *efflux*, *relocates *and *translocation*). Table 4 shows that moving from the Phrase-Chunking features to the full Protein-Transport features results in at least a 10 point gain in F-measure for both types of predicates. Interestingly, however, our additional features seem to be more helpful for Wall Street Journal predicates than GeneRIF-only predicates – Wall Street Journal predicates get a 15 point boost in F-measure, from 67.9 to 83.2, while GeneRIF-only predicates get only a 10 point boost, from 68.0 to 78.6. These results suggest that while our features are capturing many of the important characteristics of the GeneRIF domain, there may still be room for features tailored to the peculiarities of protein transport predicates.

### Analysis of model errors

To get a better idea exactly where future work on feature engineering should focus, we took a look at the mistakes our best model was making and identified a few broad classes of errors. About 40% of the model's errors could be attributed to trouble with roles that required tracing a coreference chain to find the argument. As discussed under Protein-Transport Feature in the Methods section, the scheme of [41] allows roles to be annotated in distant parts of the sentence if a coreference chain links the predicate and the distant argument. So for instance, *p53 *in Example 9 and *Daxx *in Example 10 are marked as arguments instead of the closer pronoun *its*. Our system missed the distant PATIENT roles in both of these examples.

(9) Serine 392 exerts important effects upon [_PATIENT_ p53] stability via the inhibition of its [_ORIGIN_ nuclear] [_PREDICATE_ export] mechanism.

(10) Tryptophan 521 and serine 667 residues of [_PATIENT_ Daxx] regulate its [_ORIGIN_ nuclear] [_PREDICATE_ export] during glucose deprivation

Another 20% of the errors were due to boundary mismatches, where our system predicted shorter or longer arguments. Most such errors appeared to be due to errors in our syntactic chunkers and clause chunkers, which were trained on Wall Street Journal text, not biomedical text. In Example 11, *substrate *was chunked as a verb phrase instead of as part of a noun phrase, and so our system identified only *1 *as a PATIENT, instead of the full *Insulin receptor substrate 1*.

(11) [_PATIENT_ Insulin receptor substrate 1] [_PREDICATE_ translocation] to the [_DESTINATION_ nucleus]

Finally, for about 15% of the errors it looked like having a complete syntactic parse would have helped. In these errors, one role was often separated from the predicate by something like an appositive. In Example 12, the PATIENT *protein *was missed because it was separated from the predicate by *overexpressed in prostate cancer*.

(12) This [_PATIENT_ protein], overexpressed in prostate cancer, [_PREDICATE_ shuttles] between the cytoplasm and the nucleus.

**Figure 1:**
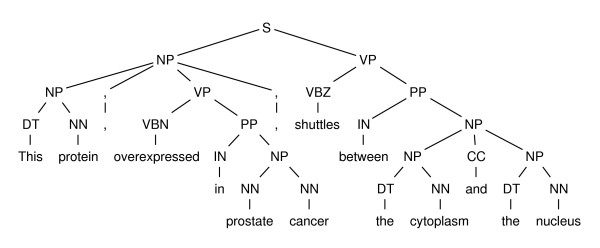
**Example syntactic tree**. This figure shows the syntactic tree for the phrase *This protein, overexpressed in prostate cancer, shuttles between the cytoplasm and the nucleus*.

Figure 1 shows that with a syntactic parse, *protein *is the head noun of the predicate's NP complement, essentially only two constituents away, compared to a distance of six words (including punctuation) when not using a syntactic parse.

These three classes of errors accounted for about 75% of the errors made by our system. They suggest that future research on protein transport roles could benefit by including features derived from coreference systems [27,42-44] to better link predicates with their distant arguments, by training some of our intermediate components like syntactic and clause chunkers on biomedical text, and by including some features extracted from full syntactic parses.

### Comparison to the OpenDMAP method

Unfortunately, we cannot directly compare the numbers here to those of OpenDMAP, which reported precision of 0.75 and a recall of 0.49 [27]. First, they evaluated only the predicate *translocation *while many other transport predicates (e.g. *import*) are considered in this work. Second, they calculated precision and recall at the sentence level instead of the individual role level, and included identifying the predicate as part of the task. Third, different data preprocessing strategies were used. For example, conjunctions in GeneRIFs were handled differently (see details in the Result Section). Although we cannot perform head-to-head comparison, we would like to point out that the two approaches are potentially complementary to one another. The OpenDMAP system is primarily based on curated ontologies and patterns that make it capable of assigning identified roles with high precision. However, automatically recognizing biomedical concepts (e.g. gene/proteins) remains challenging by current technologies. The statistical SRL system presented here provides a potential remedy because of its robustness in identifying roles in text. We are going to explore potential synergies between the two systems in the future, but this is beyond the scope of this work.

### General limitations

While the performance of our protein transport role labeling system is quite good, it is worth reflecting on a few of its limitations. First, our systems used word-chunking methods instead of constituent classification methods (and some of the benefits and drawbacks of this choice are discussed in the Methods section). In general, word-chunking models were useful for our data because they resulted in a much faster system with the ability to identify roles that were smaller than typical syntactic tree constituents. However, as noted in the Analysis of model errors section above, some distant roles may be more easily identified using a syntactic tree, and therefore may be more tractable for constituent classification methods.

Another limitation of this study was that our models were trained and evaluated on protein transport predicates, which are not fully representative of all the different predicates in biomedical text. As a consequence of this, the AGENT role was almost completely absent from our data (as discussed in the Analysis by Role Type section above). It is quite possible that other biomedical predicates use AGENT roles much more often, and the system would have to learn to better distinguish between AGENT and PATIENT. In newswire verbs, AGENT and PATIENT roles were among the easiest to identify [8,45], but since biomedical predicates are often nouns, it may be necessary to introduce some additional features to make these distinctions.

Finally, our approach showed that model performance could be improved substantially by introducing some domain specific features. However, the need for these features indicates that adapting semantic role labeling methods to the biomedical domain is more work than just building a corpus and retraining an existing model. In our case, the additional features were mostly based on biomedical part of speech and named entity (protein) tags, which we used in addition to the standard newswire tags. Thus, our results suggest that to build successful role labeling systems for new domains, not only is it necessary to create an appropriate corpus and train a new machine learning model, but it is also necessary to train new models for all subcomponents (e.g. part of speech taggers, named entity taggers, etc.) and include the outputs of these models as additional features.

Despite these limitations, the current level of performance of our semantic role labeling system provides two important facilities to the research community. First, semantic roles identified with reasonable precision can be used to increase the efficiency of manual curation efforts, by providing a small set of relevant passages that curators may consider for entry into their databases. Second, even imperfect semantic role labeling performance may be of use to downstream components that know how to integrate noisy data. For example, [46] showed that including noisy information based only on word co-occurrence statistics substantially improved the quality and coverage of protein-protein interaction networks. Thus, we expect that our semantic role labeling system, which achieved precisions as high as 87.6%, should be a useful sub-component for a variety of information extraction tasks.

## Conclusion

We have presented a model for identifying the semantic roles of protein transport predicates. A corpus was collected of GeneRIFs describing biological processes where proteins were moved from one cellular component to another. The predicates describing these processes, both nouns and verbs, were annotated for their semantic roles. This corpus then served as the basis for several machine learning experiments.

We explored word-chunking approaches to semantic role labeling, both to avoid the need for a computationally expensive syntactic parse, and because the traditional Penn TreeBank notions of syntactic constituent boundaries mismatched badly with the annotated protein transport roles. Thus, we trained support vector machine classifiers to classify words as being at the beginning, inside or outside of a protein transport role (AGENT, PATIENT, ORIGIN or DESTINATION). We trained these models using the features of previous word-chunking models, features adapted from other types of models, and features derived from analysis of our protein transport data.

In the end, our models were able to achieve a 87.6% precision and 79.0% recall using manually annotated protein boundaries, and 87.0% precision and 74.5% recall using automatic ones. Our protein transport models outperformed models trained on only the traditional word-chunking and phrase-chunking features, indicating that the new features we engineered for the biomedical domain were effective in addressing some of the domain differences. And the relatively small drop in performance when using automatically identified proteins suggests that our models are relatively robust to imperfect features, which is a necessity for working with real-world biomedical langauge processing systems. Analysis of our models suggested that future research should focus on including features derived from coreference resolution systems, improving the performance on biomedical text of components like syntactic chunkers and protein identifiers, and exploring features based on full syntactic parses.

## Methods

### Protein transport data

A set of 1218 GeneRIFs was collected from two sources: GeneRIFs from genes known to be involved in protein transport (e.g. *Src*, a tyrosine kinase playing critical roles in signaling) and GeneRIFs containing predicates known to express transport (e.g. *translocation *or *export*). The predicates in each of these GeneRIFs were annotated with the roles AGENT, PATIENT, ORIGIN and DESTINATION by domain experts following the annotation guidelines of [41] and using the Knowtator annotation tool [47]. GeneRIFs that did not express protein transport (e.g. because they expressed some other type of transport like DNA transport) were discarded, resulting in a final data set of 837 GeneRIFs.

Initially, this produced 1009 predicate annotations with 1803 labeled roles. However, in [41], if a predicate has a role containing a conjunction, e.g. *HopO1-1, HopS1, and HopS2*, the predicate would have been annotated three times, one for each conjoined element. This is a departure from most other semantic role labeling schemes, like PropBank and FrameNet, where the predicate would have been annotated only once, and the whole phrase *HopO1-1, HopS1, and HopS2 *would have been annotated as the argument. Having predicates annotated multiple times would require some substantial changes for most semantic role labeling architectures, which assume that each predicate needs to be visited only once to identify all of its roles. Thus, since the mapping from one form to the other was fairly straightforward, we decided to convert the annotations to the more widespread PropBank/FrameNet style of annotation. Table 5 shows the statistics for the resulting corpus.

**Table 5:** Corpus statistics.

	All	Train	Test
GeneRIFs	837	637	200
Words	21620	16446	5174
Unique words	3841	3249	1459

Predicates	911	693	218
Unique predicates	86	72	44
Unique predicate lemmas	34	28	25

Roles	1544	1159	385
AGENT roles	17	14	3
PATIENT roles	822	623	199
ORIGIN roles	173	128	45
DESTINATION roles	532	394	138

This table shows some basic statistics for the semantic roles annotated over the GeneRIFs in the protein transport corpus.

### Machine learning models

Traditional approaches to semantic role labeling have generally fallen into one of two classes: syntactic constituent approaches and word chunking approaches. Syntactic constituent approaches [4-9] look for predicate roles in the nodes of a syntactic parse tree, and have generally had the best performance on the standard test sets. Word chunking approaches [40,48] look for predicate roles in sequences of words, generally with somewhat lower performance than syntactic constituent approaches, but avoiding the heavy reliance on the expensive syntactic parsing process. To evaluate their appropriateness for protein transport role labeling, we consider each of these techniques in a little more depth.

Syntactic constituent approaches to semantic role labeling work by asking whether each constituent in the syntactic tree is a role or not. For example, given the sentence:

This protein, overexpressed in prostate cancer, shuttles between the cytoplasm and the nucleus

A syntactic constituent role labeler would examine the syntactic tree in Figure 1 and ask:

• Is [_DT_ This] a role of *shuttles*?

• Is [_NN_ protein] a role of *shuttles*?

• Is [_NP_ [_DT_ This] [_NN_ protein]] a role of *shuttles*?

• ...

A machine learning model is trained to answer such questions using features like the path of tree nodes between the predicate and the constituent in the tree, the voice of the predicate (active or passive), etc. The best of these systems are able to achieve precisions and recalls just above 80% [8,9]. This approach has also proven successful for identifying the semantic roles of some verbal predicates in biomedical data [15]. However, this approach requires a computationally expensive syntactic parse, and relies on the boundaries of the syntactic parse constituents matching the boundaries of the semantic roles.

Word chunking approaches to semantic role labeling avoid the need for a syntactic parse, and can be more flexible about the boundaries of semantic roles, though usually at some cost to performance [9,40,48,49]. The word-chunking formulation converts the semantic role labeling problem into a word classification problem by selecting appropriate labels for each word in the phrase. These labels are usually a combination of a B(eginning), I(nside) or O(utside) prefix that indicates the location of the word within the role, and a role suffix that indicates the type of the role containing the word. So for example, given the sentence *Sales declined 10% to $251.2 million from $258.7 million*, its words would be labeled as in Table 6. The best word chunking semantic role labelers acheived precisions and recalls around 70%, about 10 points below their constituent based counterparts, but with dramatically faster run times as no syntactic parse was required.

**Table 6:** Newswire semantic role chunk labels.

Sales	B_ARG0
declined	O
10	B_ARG2
%	I_ARG2
to	O
$	B_ARG4
251.2	I_ARG4
million	I_ARG4
from	O
$	B_ARG3
278.7	I_ARG3
million	I_ARG3
.	O

This table shows the semantic role chunk labels for the Wall Street Journal sentence *Sales declined 10% to $251.2 million from $258.7 million*.

#### Word-chunking classification models

Though both constituent-based and word-chunking approaches could conceivably be applied to our protein transport data, we adopted the word-chunking approach for two reasons. First, a straightforward constituent based approach would have a maximum recall of 80% for our data set as 20% of our roles did not align to constituents. A word-chunking model does not have such a cap on its recall as it is not restricted by the TreeBank notion of phrase structure. (There may be ways to address this problem in the constituent based approach, but they would require substantial new extensions to the basic syntacitc constituent paradigm.) Second, while there is ongoing work to adapt syntactic parsers to biomedical text [50,51], performance is still much lower than on newswire text. Thus it is useful to determine how far protein transport role labeling can get without relying on a syntactic parse.

The basic word-chunking formulation of semantic role labeling looks much the same on our protein transport data as it did on newswire text. So, for example, in trying to identify the roles of *translocation *in the phrase *to induce the nuclear translocation of NF-kappaB transcription factor*, we attempt to label the words as in Table 7. The word *nuclear *begins (and ends) a DESTINATION role, the word *NF-kappaB *begins the PATIENT role that *transcription *and *factor *are inside of, and all other words are outside of protein transport roles.

**Table 7:** GeneRIF semantic role chunk labels.

to	TO	O
induce	VB	O
the	DT	O
nuclear	JJ	B_DESTINATION
translocation	NN	O
of	IN	O
NF-kappaB	NN	B_PATIENT
transcription	NN	I_PATIENT
factor	NN	I_PATIENT

This table shows the semantic role chunk labels for the GeneRIF phrase *to induce the nuclear translocation of NF-kappaB transcription factor*. The table also includes Penn TreeBank style part of speech tags for each word, and identifies what a one-word feature window for the classification of the word *transcription *looks like.

To train on such data, machine learning models try to assign to each word its corresponding chunk label. Of course, doing so requires some knowledge of the context (e.g. "have I already started a role or am I outside of one?") and so, as is common for word-chunking approaches, we considered a window around the word being classified. This meant that to classify a single word, the machine learning algorithm looked at both the features for that word, and the features and labels of some preceding and following words. For example, given the word and part of speech as features, and a window of one word on either side, the word *transcription *would be classified using the window of features and labels outlined in Table 7. Note that this windowing strategy allows the algorithm to recognize that when it sees the word *transcription *it is already inside of a PATIENT role. This kind of context is crucial for high quality word-chunking. We used an existing word-chunking package, YamCha [39], to train our models. YamCha is based on Support Vector Machine models and has performed well on a variety of similar tasks [39,40].

#### Word-chunking features

Of course, as in all machine learning problems, selecting an appropriate set of features for the task plays a critical role in the success of the algorithm. We began with the basic features used in the word-chunking model of [40], omitting only the features for people, organizations and locations which generally do not occur in GeneRIF data. This resulted in the feature set:

• The text of the word

• The text of the predicate

• The part-of-speech (POS) of the word

• The BIO tag for the phrase that includes the word

• The brace tag indicating how many clauses start and end at the word

To get a better idea of how these features work, consider the sentence:

(13) BARD1 induces BRCA1 intranuclear foci formation by increasing RING-dependent [_PATIENT__1 _BRCA1] [_DESTINATION__1 _nuclear] [_PREDICATE__1 _import] and inhibiting [_PATIENT__2 _BRCA1] [_ORIGIN__2 _nuclear] [_PREDICATE__2 _export]

Table 8 shows the features for a few of the words in the sentence when the roles of the predicate *import *are being identified. Producing such feature values relied on the output of some existing sub-components:

**Table 8:** Features for Example 13.

Word	Predicate	POS	Phrase	Clause
by	import	IN	B-PP	*
increasing	import	VBG	B-VP	(*
RING-dependent	import	JJ	B-NP	*
BRCA1	import	NNP	I-NP	*
nuclear	import	JJ	I-NP	*
import	import	NN	I-NP	*
and	import	CC	O	*
inhibiting	import	VBG	B-VP	*
BRCA1	import	JJ	B-NP	*
nuclear	import	JJ	I-NP	*
export	import	NN	I-NP	*)

This table shows the basic word-level features that were used to characterize each word in Example 13.

• Word stems are determined by a lookup table from the University of Pennsylvania of around 300,000 words [52].

• Part-of-speech tags are identified by the MXPOST part-of-speech tagger [53].

• Syntactic phrases are determined by a YamCha-based chunking system trained on the CoNLL 2000 [54] text chunking data

• Clause boundaries are determined by a YamCha-based chunking system trained on the CoNLL 2001 [55] clause identification data.

All of these sub-components were simple surface level processors that considered only things like the words themselves and orthographic features like capitalization and punctuation.

#### Phrase-chunking features

This basic set of features was small however, and missed some important characteristics of the task. To augment our feature space, we turned to Hacioglu et. al.'s phrase-chunking model [40]. The model itself was inappropriate for our task because, just as the syntactic constituent classification models, the phrase classification model considered phrases whose boundaries often did not align with our roles. The model's features, however, had a relatively straightforward translation to word-level features instead of phrase-level features and thus we modified them in that way for use with our model. To explain these new features, we again refer to the sentence from Example 13, repeated here as Example 14:

(14) BARD1 induces BRCA1 intranuclear foci formation by increasing RING-dependent [_PATIENT__1 _BRCA1] [_DESTINATION__1 _nuclear] [_PREDICATE__1 _import] and inhibiting [_PATIENT__2 _BRCA1] [_ORIGIN__2 _nuclear] [_PREDICATE__2 _export]

The phrase-chunking features included all of the word-chunking features and the following additional features:

• 2, 3, and 4 character suffixes of the word, e.g. the suffixes for *nuclear *are *-ar*, *-ear *and *-lear*.

• The stem of the predicate, e.g. the stem of *imports *would be *import*

• The part-of-speech (POS) of the predicate, e.g. the POS of *nuclear *is JJ

• The number of predicates in the sentence, e.g. there are 2 predicates, *import *and *export*.

• The part-of-speech of the word before the predicate, e.g. the predicate *import *is preceded by the part-of-speech JJ (the POS of the word *nuclear*).

• The part-of-speech of the word after the predicate, e.g. the predicate *import *is followed by the part-of-speech CC (the POS of the word *and*).

• The two phrase types preceding the predicate, e.g. the noun phrase including *import *is preceded by a PP (prepositional phrase), *by*, and a VP (verb phrase), *increasing*.

• The two phrase types following the predicate, e.g. the noun phrase including *import *is followed by a VP (verb phrase), *inhibiting*, and a NP (noun phrase), *BRCA1 nuclear export*.

• The location of the word relative to the predicate, e.g. *nuclear *is BEFORE the predicate *import *and *inhibiting *is AFTER the predicate.

• The distance between the predicate and the word in number of phrases, e.g. *inhibiting *is 1 phrase away (1 VP) from the predicate *import*, and *export *is 2 phrases away (1 VP and 1 NP) from the predicate.

• The distance between the predicate and the word in number of verb phrases, e.g. both *inhibiting *and *export *are 1 VP away from the predicate *import*.

• The phrasal path between the predicate and the word, e.g. the path from the second *BRCA1 *to *import *is NNP < NP < NN (it is in the same NP as *import*), and the path from *import *to *inhibiting *is NN>NP > VP > VBG (it is one NP and one VP away from *import*).

• The clause boundaries between the predicate and the word, e.g. there is a single open clause boundary, '(', between *by *and the predicate *import*.

• The clause boundaries between the sentence boundary and the word, e.g. there are two open clause boundaries, '((', between *BRCA1 *and the beginning of the sentence.

#### Protein-transport features

Preliminary experiments (carried out as cross-validations on the training data) showed that our models were having difficulties with a few different areas of our data: the boundaries of protein names, conjoined predicates and arguments tied to a predicate through coreference.

We noticed early on that our models were having trouble determining when a phrase immediately preceding a predicate should be identified as a PATIENT. For example, our early models identified *GLUT4 requires *instead of *GLUT4 *as the PATIENT in Example 15, and couldn't find any PATIENT at all in Example 16.

(15) These results suggest that [_PATIENT_ GLUT4] requires [_PREDICATE_ translocation]...

(16) ... involved in [_PATIENT_ eNOS] [_PREDICATE_ translocation]...

The system had learned a strategy that identified as the PATIENT everything from the last "proper noun" up to the predicate. In these two examples, the part-of-speech tagger identified only GLUT4 as a proper noun, and so not only did the system incorrectly include *requires *as part of the PATIENT in Example 15, but it also failed to include the PATIENT *eNOS *in Example 16. These errors indicated that our models were having trouble identifying the boundaries of protein names.

Our models were also having trouble with conjoined predicates, particularly when an argument was present for the first but elided for the second. So, for instance, in Example 17, *protein *is the PATIENT of both *folding *and *translocation*, and in Example 18, *Tir *is the PATIENT of both *secretion *and *translocation*. In both of these examples, our early models failed to identify *protein *and *Tir *as PATIENT roles of the *translocation *predicates.

(17) ... for ERdj5 in [_PATIENT_ protein] folding and [_PREDICATE_ translocation]...

(18) ... for efficient [_PATIENT_ Tir] secretion and [_PREDICATE_ translocation]...

Though our models were given a window of features around the word classified, this window was generally no more than two words before of after the word. (We experimented with larger windows, but these models only performed worse.) Thus words like *protein *and *Tir *above were too distant from the predicate to be considered as arguments, and so our models failed on them.

Finally, our models had trouble with the annotation style of [41] in that it annotates some roles that are tied to the predicate only through a coreference chain. Example 19 shows such a role.

(19) a rapid activation of the [_PATIENT_ acid sphingomyelinase] correlating with its microtubule- and microfilament-mediated [_PREDICATE_ translocation]

In this example, the predicate *translocation *is contained within the prepositional phrase *with its... translocation*. PropBank-style annotation would thus likely annotate *its *as the PATIENT of *translocation*. However, the annotation style of [41] allows for implicitly following up the coreference chain to conclude that *its *actually refers to *acid sphingomyelinase*, and then annotating *acid sphingomyelinase *as the PATIENT instead. These sorts of annotation decisions typically distance the argument from its predicate and make it difficult for our system to find the role.

To address these three issues – unidentified proteins, conjoined predicates and coreference chains – we introduced the following additional features:

• A set of orthographic features that capture some of the irregularities of protein names. These included:

- The capitalization class of the word; one of INITIAL-UPPER, ALL-UPPER, ALL-LOWER, MIXED-UPPER-LOWER or OTHER

- The numeric class of the word; one of YEAR-DIGITS, DIGITS, ALPHANUMERIC, SOME-DIGITS, ROMAN-NUMERAL or OTHER

- The punctuation class of the word; one of PUNCT-ONLY, INITIAL, POSSIBLE-INITIAL, ACRONYM, or HAS- plus one or more of DOT, DASH, SLASH or COMMA for each contained in the word.

• The part of speech tag output by MedPost [56], a part of speech tagger trained on biomedical data, and therefore less likely to perform poorly when encountering protein names.

• A protein BIO-chunk label of the word, i.e. B_PROTEIN, I_PROTEIN or o. We examined both manually annotated proteins, to give us an idea of the maximum possible performance, and proteins annotated automatically by ABNER [57], a model based on conditional random fields and orthographic and gazetteer-based features.

• A feature indicating whether or not the word was in a base-phrase conjoined with the base-phrase of the predicate, and which conjunction was conjoining them, e.g. *and *or a comma.

• A feature indicating whether or not the word was part of the last protein before a pronoun. This is essentially a poor-man's coreference resolution scheme.

In combination with the Word-Chunking features and the Phrase-Chunking features discussed above, these features served as the basis for all our machine learning experiments on our GeneRIF protein transport data.

## Authors' contributions

SB and ZL wrote most of this paper. SB designed and trained the machine learning models, and performed the experimental evaluations. ZL collected the GeneRIF data and participated in the experimental designs. JHM and LH helped to guide and advise the project. All authors read and approved the final manuscript.

## Appendix: list of protein transport role examples

The following are all the examples of protein transport predicates and their roles given in the article.

(1) [_PATIENT_ Bax] [_PREDICATE_ translocation] from the [_ORIGIN_ cytosol] to [_DESTINATION_ mitochondria] leads to the subsequent formation. ...

(3) [_PREDICATE(NOUN)_ Truncation] of up to 44 C-terminal amino acids from the putatively cytoplasmic C-terminal hydrophilic domain left transport function [_PREDICATE(ADJ)_ unimpaired], but [_PREDICATE(NOUN)_ deletion] of the adjacent STAS (sulfate transporter anti-sigma factor antagonist) domain [_PREDICATE(VERB)_ abolished] function.

(4) IRS-3 expression blocked glucose/IGF-1 induced [_PATIENT_ IRS-2] [_PREDICATE_ translocation] from the [_ORIGIN_ cytosol] to the [_DESTINATION_ plasma membrane].

(9) Serine 392 exerts important effects upon [_PATIENT_ p53] stability via the inhibition of its [_ORIGIN_ nuclear] [_PREDICATE_ export] mechanism.

(10) Tryptophan 521 and serine 667 residues of [_PATIENT_ Daxx] regulate its [_ORIGIN_ nuclear] [_PREDICATE_ export] during glucose deprivation

(11) [_PATIENT_ Insulin receptor substrate 1] [_PREDICATE_ translocation] to the [_DESTINATION_ nucleus]

(12) This [_PATIENT_ protein], overexpressed in prostate cancer, [_PREDICATE_ shuttles] between the cytoplasm and the nucleus.
